# HOW TO CHOOSE THE RIGHT JOURNAL FOR YOUR MANUSCRIPT

**DOI:** 10.1093/geroni/igz038.820

**Published:** 2019-11-08

**Authors:** Laura P Sands

**Affiliations:** Virginia Tech, Blacksburg, Virginia, United States

## Abstract

Article submissions to The Gerontological Society of America’s high impact scientific journals continue to increase each year which has led to editors becoming more selective about which articles are accepted for publication. The purpose of this session is to describe how to efficiently and successfully navigate the process of determining which journal is the best fit for your manuscript. The specific objectives are to: (1) increase understanding the differences in scope and features of each journal; (2) inform how to determine which journal is most appropriate for the topic and methods of your manuscript; and (3) describe how to convey the scientific contribution of your article to the journal.

